# Sex Differences in Autism Spectrum Disorder: Diagnostic, Neurobiological, and Behavioral Features

**DOI:** 10.3389/fpsyt.2022.889636

**Published:** 2022-05-13

**Authors:** Antonio Napolitano, Sara Schiavi, Piergiorgio La Rosa, Maria Camilla Rossi-Espagnet, Sara Petrillo, Francesca Bottino, Emanuela Tagliente, Daniela Longo, Elisabetta Lupi, Laura Casula, Giovanni Valeri, Fiorella Piemonte, Viviana Trezza, Stefano Vicari

**Affiliations:** ^1^Medical Physics Department, Bambino Gesù Children's Hospital, IRCCS, Rome, Italy; ^2^Section of Biomedical Sciences and Technologies, Science Department, Roma Tre University, Rome, Italy; ^3^Division of Neuroscience, Department of Psychology, Sapienza University of Rome, Rome, Italy; ^4^Neuroradiology Unit, Imaging Department, Bambino Gesù Children's Hospital, IRCCS, Rome, Italy; ^5^NESMOS, Neuroradiology Department, S. Andrea Hospital Sapienza University, Rome, Italy; ^6^Head Child and Adolescent Psychiatry Unit, Neuroscience Department, Bambino Gesù Children's Hospital, IRCCS, Rome, Italy; ^7^Neuromuscular and Neurodegenerative Diseases Unit, Bambino Gesù Children's Hospital, IRCCS, Rome, Italy; ^8^Child Neuropsychiatry Unit, Neuroscience Department, Bambino Gesù Children's Hospital, IRCCS, Rome, Italy; ^9^Life Sciences and Public Health Department, Catholic University, Rome, Italy

**Keywords:** ASD, gender, animal models, imaging, neurobiological mechanism

## Abstract

Autism Spectrum Disorder (ASD) is a complex neurodevelopmental disorder with a worldwide prevalence of about 1%, characterized by impairments in social interaction, communication, repetitive patterns of behaviors, and can be associated with hyper- or hypo-reactivity of sensory stimulation and cognitive disability. ASD comorbid features include internalizing and externalizing symptoms such as anxiety, depression, hyperactivity, and attention problems. The precise etiology of ASD is still unknown and it is undoubted that the disorder is linked to some extent to both genetic and environmental factors. It is also well-documented and known that one of the most striking and consistent finding in ASD is the higher prevalence in males compared to females, with around 70% of ASD cases described being males. The present review looked into the most significant studies that attempted to investigate differences in ASD males and females thus trying to shade some light on the peculiar characteristics of this prevalence in terms of diagnosis, imaging, major autistic-like behavior and sex-dependent uniqueness. The study also discussed sex differences found in animal models of ASD, to provide a possible explanation of the neurological mechanisms underpinning the different presentation of autistic symptoms in males and females.

## Introduction

One of the most consistent data in Autism Spectrum Disorder (ASD) is the higher prevalence in males compared to females (1). According to the fifth edition of the Diagnostic and Statistical Manual of Mental Disorders (DSM-5), the term “Autism Spectrum Disorder” refers to a neurodevelopmental condition emerging early in life characterized by impairments in social interaction and communication, associated with differences in sensory processing as well as restricted and repetitive behaviors, interests and activity (2). The most recent estimates of ASD prevalence in United States are 23.0 per 1,000 (one in 44) children aged 8 years, and ASD was 4.2 times as prevalent among boys as among girls (1). The combined male-to-female prevalence ratio was 4.3:1; site-specific ratios ranged from 3.4:1 to 4.7:1, with little evidence of heterogeneity by site (1). This finding is in line with DSM-5 states that ASD is diagnosed four times more often in males than in females and this is also confirmed by 2010 Global Burden of Disease study (3) that reported an estimate of 4:1. This review is principally focused on looking into the most significant studies that investigated differences in ASD males and females to shed some light on the peculiar characteristics of this prevalence in terms of diagnosis, clinical manifestations, psychiatric comorbidity, brain imaging and neurobiological features. Moreover, the review discusses sex differences in animal models of ASD, to explore the neurobiological mechanisms underpinning the different presentation of autistic symptoms in males and females and the sex-dependent uniqueness. The aims of this review are to provide an update on sex difference in ASD, by (1) analyzing sex ratio in epidemiological studies; (2) comparing and analyzing the heterogeneity of manifestations of core symptoms and psychopathological comorbidities between males and females; (3) providing a possible explanation of the neurobiological mechanisms underpinning the different presentation of autistic symptoms in males and females, and (4) summarizing sex differences found in animal models of ASD.

## Sex Difference in Epidemiological Studies

The four-to-one sexratio mentioned above is broadly cited and comes from research studies that ascertained the mean male-to-female ratio from population prevalence studies of ASD. However, sex ratio in prevalence is still debated and recent epidemiological administrative and community-based studies have reported ratios ranging from 2:1 to 5:1 (4, 5). The assumption that ASD is more commonly diagnosed in males than in females has motivated significant theories about the nature and etiology of ASD: the Extreme Male Brain (6), Female Protective Effect (7–9), and Female Autism Phenotype theories (10–12). The Extreme Male Brain Theory suggest that the two dimensions for understanding human sex differences are “empathising” and “systemising.” According to this, the male brain is defined as the one in which systematization fits better than empathy. The female brain, on the other hand, is defined with an opposite cognitive profile. Using these definitions, ASD can be considered as an extreme of the normal male profile (6). Increased male prevalence has been also repeatedly reported leading to the concept of a “Female Protective Model/Effect.” This model assumes that risk for ASD is quantitative, that it follows a distribution in the general population, and that females are protected from the impact of becoming autistic (9). Female protective effect leads to a reduced prevalence of ASD compared with males with male-to-female ratio of 7:1 for high-functioning ASD to 2:1 for individuals with moderate to severe Intellectual Disability (7, 8, 13). Faced with risk factors, females seem to be protected from becoming autistic and the risk burden threshold that females must carry (e.g., genetic variants) or experience (e.g., environmental exposures), before their ASD became evident is greater than for males (9). Moreover, Female Autism Phenotype theories suggest the presence of a female-specific manifestation of autistic strengths and difficulties, which fits imperfectly with current, male-based conceptualisations of ASD (2, 14–16). There is evidence to support the existence of the female ASD phenotype. For example, there is empirical evidence that girls and women with ASD show greater social motivation and a greater capacity for friendships than males with ASD (10–12). However, the higher prevalence in males has been questioned several times in light of various factors and simple averages of sex ratios may not capture a key feature of ASD; also epidemiological studies with similar inclusion criteria and recruitment methods demonstrated wide variability in ASD sex ratios ranging between eight-to-one and two-to-one (17). Recent meta-analyses are useful as an overview to the male-to-female ratio in ASD, although some researches do not take into account the methodological quality of the study, especially regarding case ascertainment methods such as Active vs. Passive case-finding methods (4). Studies that *actively* searched for cases of ASD, regardless of whether they had already been identified by clinical or educational services, tended to identify more females with ASD than *passive* studies, which only detect cases if they have already been diagnosed by clinical or educational services. The results of the meta-analysis of Loomes et al., showed that only when considering the studies with the highest methodological quality and those using active case- ascertainment methods, the male-female odds ratios were lower and there was consistency between the studies, with no significant heterogeneity observed. In light of this, the male-to-female ratio of 4 to 1 is likely inaccurate and more accurate male-to-female ratio for ASD is <3.5 to 1 (4). Despite this, the bias in favor of males is confirmed and underlines the importance of investigating possible differences in terms of clinical manifestations and etiology.

## Sex Difference in Core Symptoms

The clinical presentation of ASD symptoms can be dissimilar in males and females individuals (10, 14, 18, 19). Additionally, despite the fact that the tools and techniques for assessment have been refined over time, a portion of girls with higher cognitive and language abilities are at risk of not being identified until later in life (18, 20–22). A population-based study in the UK showed that females with similar levels of symptom expression to males were less likely to receive a diagnosis of ASD from clinical services (23). Moreover, boys are usually detected by ASD screeners at higher rates than girls, although sex differences in screening scores are not as pronounced (24, 25). Recent studies on sex differences in ASD symptomatology often show contrasting results and appear to vary across age groups and symptom domains. For example, diagnosing ASD during adulthood may be difficult for clinicians, for several reasons. Possible challenges are related to the difficulties encountered in gaining information about the developmental history and the presence of coping and camouflaging strategies (26). Adults with ASD may have never referred to child or adult psychiatric services (i.e., missed diagnoses), they may have been incorrectly diagnosed with other psychiatric disorders during life and/or the co-occuring psychiatric disorder could have partially cover ASD core symptoms. Adults females and males with ASD usually have first access to Mental Health Services for social problems, feelings of anxiety and mood disturbances. The most common earlier diagnoses were anxiety and mood disorders or psychosis-related disorders. The risk of going undiagnosed is even more elevated for women. Females are frequently diagnosed later than their male peers and this is possibly related also to standardization of diagnostic tools on male samples. Moreover, women usually present more internalizing than externalizing symptoms, which might be easily confused with anxiety or depression and may not be noticed (26). Regarding difference in core symptoms adult females with ASD also reported significantly higher scores than men in the Hyper/Hyporeactivity to sensory input domain specifically among subjects who were misdiagnosed (27). The presence of sensory profile abnormalities among the most recent diagnostic criteria may lead to an improved recognition rate of females with ASD (28). During childhood and adolescence some studies have shown that females with ASD are less likely to show overt patterns of limited and restricted interests than males. Furthermore, considering the age developmental trajectories, males and females with ASD before age of 4 seem to show no gender differences in core symptoms (29, 30); besides, sex does not seem to relate to the possibility of receiving an earlier diagnosis (31, 32). Moreover, the frequency of regression (i.e., loss of previously learned language, motor, or other skills, occurring around the age of 12 months) appears to be the same in boys and girls with ASD (33, 34). In general, the age trajectory of core symptoms in children with ASD does not appear to vary by sex (35, 36). The exceptions are repetitive behaviors and limited and restricted interests, which are more common in males over 6 years of age (37). Further, there is some evidence that females display limited and repetitive behaviors and interests that differ from males. Most males are fascinated by toys on wheels or screen time (e.g., video games), while girls mostly show obsessions with random objects (e.g., stickers, stones, pens, animals) and play obsessive and repetitive games with other toys (16, 38). Differences in core ASD symptoms may become more pronounced as individuals age and cultural influences play a larger role into gender differences (23) leading to inconsistent and conflicting results. In addition, the difference in core symptoms could be also related to the change in intervention strategies that occur between toddlerhood to preschool-school age period partly due to transition from “early home based” intervention to “school-based” (23). Also camouflaging is often discussed in relation to sex difference and offers a partial explanation of increased rates of missed or delayed diagnosis. Results from a recent meta-analysis suggest that research studies that have used qualitative methodologies were not suggestive of sex or gender differences in camouflaging (39–41). However, results of studies with psychometrically rigorous methods of quantification (i.e., continuous rating scales) generally supported sex differences (42). When examining sex differences in camouflaging frequency and pervasiveness autistic females, compared to males, reported camouflaging more frequently and across more situations (43).

It is important to consider that, studies investigating differences between male and females with ASD had some limitations included modest sizes of female, and some studies do not considered cognitive and developmental abilities (IQ) which are necessary to best compare the two groups (44). The effects of IQ and gender/sex on measures of ASD symptoms still have to be well-documented, and previous studies failed to consider multiple developmental variables or have not accounted for these factors simultaneously (45, 46). Another factor, which may contribute to the heterogeneity of results, is linked to the type of variables taken into account for core symptoms. In fact, a large variability in the results of studies using broad construct has been reported (47). Broad constructs such as DSM-5 criteria, “deficits in social interaction and communication” and diagnostic tools such as ADOS-2 (48) and ADI-R (49), can define ASD in an abstract way and can provide some evidence that cut-offs may not always be useful (22). Gender/sex differences may not be detected using broad constructs and this could potentially contribute to the under-recognition of ASD in females (50, 51). Differently, studies that use narrow constructs (e.g., peer relationship; social attention; interpersonal motor synchrony; peer engagement behaviors; play behaviors**;** difficulty engaging in back and forth conversations, use of atypical gaze, and specific types of anxiety symptoms) could be more useful to highlight specific differences. Studies have found that females with ASD display greater engagement with peers on the playground (52), greater social motivation (11), greater social reciprocity (53), and showed increased use of pragmatic social communication (54). Friendships can also be experienced differently with ASD females which are more likely to be neglected by peers than ASD males, who are more likely to be rejected (55).

## Gender/Sex Difference in Psychopathological Comorbidities

The prevalence of psychiatric comorbidity in ASD was also documented by different studies (14, 56) which detected that about 70% of individuals with ASD have behavior problems and psychiatric comorbidity. In addition, 41% of children and adolescents with ASD had two or more co–occurring disorders and more than a third had three or more disorders in addition to ASD. Specifically, the most frequent psychiatric disorders encountered are Social Anxiety Disorder (29%), Attention–Deficit/Hyperactivity Disorder (28%), Oppositional Defiant Disorder (28%), Major Depressive Disorder (0.9%), Dysthymic Disorder (0.5%), and Conduct Disorder (3%) (56). An Italian study (57) recruited a large number of children and adolescents with ASD and assessed psychopathological comorbidities using the Child Behavior Checklist—CBCL (58), showing that ~30% exhibited internalizing problems and 6% manifested externalizing problems (57). Some studies described that children and adolescent with ASD have more access to Emergency Departments (ED) than children and adolescent without ASD (59–62). Anyway, both females and males with ASD are likely to receive a diagnosis of mood disorders, behavioral disturbances, relationship problems, and abuse less frequently than other children and adolescents (61). Sex differences in psychopathological comorbidity in children with ASD is still debated and studies are still inconsistent (10, 57, 63, 64). Overall, compared to females, males with ASD demonstrated more externalizing behavior than females, such as aggressiveness, hyperactivity. Conversely, females with ASD were more likely to experience internalizing problems, depression, higher risk of suicide, anxiety and other emotional problems (52, 61, 65). However, studies still have different and contrasting results and, for example, Frazier et al. found more externalizing behavior problems, irritability, lethargy and self-injurious behaviors in females than in males (66) while, other studies found higher rate of psychiatric comorbidities in males than in females (57, 67, 68). Also some studies that have used the DSM oriented scale of Children Behavioral Checklist—CBCL (58), found no significant difference between males and females (69). When looking at hyperactivity and inattention, May et al. found sex differences in males and female with ASD: younger males with ASD were more impaired than younger females with ASD, also compared to TD male and females (70). Hull et al. detected that females with ASD showed lower Attention Deficit and Hyperactivity Disorder (ADHD) scores than males (71). Also Salazar et al. pointed out that males exhibit higher rates of ADHD and Oppositional Defiant Disorder (ODD) compared to females in children with ASD aged from 4.5 to 9.8 years with and without intellectual impairment yielded some authors to consider sex as a probable protective factor for externalizing problems (72). Age trajectory of psychopathological comorbidities can show some differences in males and females: during early adolescence ASD females demonstrating higher levels of depressive symptoms than either ASD males or TD females through parent- and self-report questionnaires. During late adolescence, ASD males and females were found to have similar levels of depressive symptoms, although males seem to have an increase in symptoms along time. With respect to anxiety, ASD females had higher levels of anxiety than ASD males in early adolescence. During late adolescence, both ASD males and females reported higher levels of anxiety compared to TD (73). Moreover, females often had a previous clinical history of multiple diagnoses: depression, anxiety, anorexia nervosa and emergence of personality disorder (63, 74). During adulthood, individuals with ASD have increased rates of major psychiatric disorders including depression, anxiety, bipolar disorder, obsessive–compulsive disorder (OCD), schizophrenia, and suicide attempts. Women with ASD were diagnosed more often with respect to men with anxiety, bipolar disorder, dementia, depression, schizophrenic disorders, and suicide attempts. Men, on the other hand, are more likely to suffer from OCD, ADHD, alcohol abuse, drug abuse, and drug dependence (75).

In summary, the most recent epidemiological and clinical studies have confirmed male predominance in ASD prevalence, sex difference in clinical manifestations and the difficulties in diagnosing females. Most of the clinical, neurobiological and preclinical studies have been focused on males (19, 76). It is our opinion that the studies should be conducted to both sexes and using human and animal models in order to enhance the validity of neurobiological hypothesis to contribute to sex-oriented prevention, diagnosis and treatment (77).

## Gender Differences and Brain Imaging

Magnetic Resonance Imaging (MRI) is nowadays a very powerful tool to study and understand complex conditions, such as ASD, especially by the exploration of microscopic anatomical features such as gray matter and white matter volumes, cortical thickness and diffusion tensor imaging parameters. In line with other investigation methods, MRI imaging itself has been attempting to find an explanation about the differences between genders in ASD (78). Given the relatively small number of females developing ASD, there are still very few studies focusing on the differences between males and females in structural and functional brain characteristics as a direct consequence or possible cause of ASD. Compared to other methods, neuroimaging provides information about the final effect that multiple etiological mechanisms contribute to generate. However, these observations may provide a better comprehension of the physiopathological basis underlying this complex disorder. MRI studies and findings may be classified in two main categories: structural and functional changes. Generally, studies on structural changes attempt to investigate both gray and white matter volume looking across the whole brain and identifying areas statistically different between the two groups. In addition, studies can be also focused on the volume of brain structures regardless gray and white matter content. Some evidence supports the hypothesis that the brain in children with ASD undergoes an abnormal growth trajectory with a period of early overgrowth and a first deep differentiation between boys and girls occurs right in the age range of 2–5 years. A study on gray and white matter volumes of 9 girls and 27 boys with an age range of 2–5 years (79) demonstrated that girls share almost the same areas of size-related abnormalities observed in males compared to healthy controls. Furthermore, additional sites of abnormality were exclusively observed in the female population, including enlargement in temporal white and gray matter volumes and reduction in cerebellar gray matter volume. Similar findings with the same number of female patients were observed in a longitudinal study (80) whereby the analysis revealed that large regions (total cerebral gray and white matter, frontal gray matter, temporal gray matter, cingulate gray matter, and parietal gray) showed an abnormal growth in ASD patients and that this abnormal growth profile was more pronounced in females than in males. A more robust evidence on the topic was given by the multicenter Autism Brain Imaging Data Exchange (ABIDE, http://fcon_1000.projects.nitrc.org/indi/abide/) initiative who recruited a large dataset of over 500 individuals with ASD. Two studies (81, 82) exploiting this dataset and including 36 and 47 females with ASD, respectively, reported a specific gender difference only when considering age into account. In particular, bilateral inferior and middle temporal lobes showed an effect of diagnosis and gender mainly in the age range of 12–14 years (81). Differently, the study of Zhang (82) showed that gray and white matter, and hippocampus volumes were larger in adult and adolescent males with ASD compared to controls, but such a difference was absent in females. In addition, female adolescents and adults with ASD had smaller right putamen volume than female controls, while there were no differences in men with ASD. The study of Schaer et al. analyzed, from the ABIDE dataset, verbal performance and IQ of 53 females with ASD within a wide age range (8–39 years) comparing them with a sample of controls matched for age. Authors showed that in a factorial design with diagnosis, gender and interaction between them, diagnosis did not have a significant main effect on cortical volume, thickness, or local gyrification. Furthermore, the gyrification of the ventromedial and orbitofrontal prefrontal cortices was only decreased in males with ASD compared to controls, whereas females seemed to have rather an increase that did not reach statistical significance (83). To date, very little is still known for females concerning the effect of rebalance seen in males. The large cortical overgrowth occurring in very early age in autistic males seems to be reduced in the period of adolescence. In males, the phase of substantial changes on cortical development in ASD is indeed moderated during the following phases, reducing the thickening in early adulthood. The same effect of age is not seen in females, mostly due to insufficient dataset power to formally test for the moderating role of age on sex/gender-differential neuroanatomy in ASD. A further evidence of gender differences in ASD was seen in a study of Nordahl et al. where an early overall brain volume overgrowth was evident in some preschooler boys with regressive ASD and absent in girls. However, the same group interestingly showed also that corpus callosum differed in size and structure in both boys and girls relative to age-sex matched controls (84). Callosal organization was evaluated using both diffusion tractography to define subregions based on cortical projection zones and midsagittal area analysis. In a sex-specific comparison with the control group, both males and females with ASD had smaller regions dedicated to fibers projecting to superior frontal cortex. However, differences between males and females were found since the former had a smaller callosal region dedicated to the orbitofrontal cortex and the latter had smaller callosal region to the anterior frontal cortex. A recent large multinational sample from the Enhancing Neuroimaging Genetics through Meta-Analysis (ENIGMA) ASD working group comprised 1,571 patients with ASD (224 females) and 1,651 healthy control subjects (age range, 2–64 years). The authors (85) found no evidence of a sex-by-diagnosis interaction and conclude that the increased volumes and thickness in both males and females with ASD could be taken as evidence for the “extreme male brain” hypothesis (6), where human maleness is strongly related to ASD. This hypothesis has been supported by a study of Ecker et al. that developed a predictive model of biological sex based on cortical thickness (86). The study was performed on 98 individuals with ASD (49 females and mean age = 23 years) and it examined the probability of the disease as a function of normative sex-related phenotypic diversity in brain structure. In particular, 68.1% (32 of 47) of all biological female individuals were correctly allocated to the category of phenotypic female individuals and 74.5% (38 of 51) of all biological male individuals to the category of phenotypic male individuals. Conversely, 39 of 49 female individuals (79.6%) were allocated to the category of phenotypic male individuals. No such differences were observed in male individuals, who were correctly allocated to the male category in 81.6% (40 of 49) of all cases. Based upon these findings, the authors conclude that female individuals with a more male-typical pattern of brain anatomy are significantly more likely to have ASD compared to female individuals with a characteristically female brain phenotype.

The idea that the overall female pattern of ASD-related brain changes can be a resembling to neuronal masculinization seems to have evidence not only in morphological patterns (87), but also in brain functioning and connectivity. Considering the fact that healthy males normally show higher functional connectivity compared to healthy females, a study on resting state fMRI (88) was able to prove how females with ASD tend to present a pattern of hyperconnectivity when compared to the same gender control group. The hypothesis of this female masculinization process in ASD is more supported compared to a male feminization one. Conversely, the mentioned above study by Di et al. with resting-state functional MRI data (28 autistic females and 129 autistic males) looked at functional connectivity differences between male and females with ASD showing different patterns (81). Functional connectivity among 153 regions across whole brain showed a diagnosis by sex interaction in the connectivity between the precuneus and medial cerebellum as well as the precunes and dorsal frontal cortex. While males with ASD presented higher connectivity in these connections compared with healthy males, females with ASD had lower connectivity. A study on large-scale resting state fMRI samples, from the open-access ABIDE database (760 healthy males and 471 and 360 males with ASD), partially tested the hypothesis of the Gender Incoherence (GI) model (89). GI predicts a shift-toward maleness in females, yet a shift-toward-femaleness in males with ASD. Across all resting state fMRI metrics, results revealed coexisting, but network-specific, shift-toward-maleness and shift-toward-femaleness in males with ASD. A shift-toward-maleness mostly involved the default network, while a shift-toward-femaleness mostly occurred in the somatomotor network. Similarly the fMRI from ABIDE dataset was used by Tavares et al. by reporting as principal findings an ASD female-specific altered connectivity involving visual, language and basal ganglia networks, in line with ASD cognitive and neuroscientific theories (90). Another recent study reported the use of fMRI from the ABIDE in the implementation of a novel explainable artificial intelligence (XAI)-based framework, using also deep neural networks (DNN), to investigate neurological principles of ASD (91, 92). In particular, authors developed a novel spatiotemporal DNN model to learn functional brain organization patterns that could distinguish between the two gender groups. Main findings indeed reported the identification of functional brain features, especially between the primary motor cortex and supplementary motor area that clearly distinguished between males and females with ASD. Moreover, the analysis conducted by Supekar et al. also identified bilateral middle and superior temporal gyri as brain areas whose features clearly distinguished between the two groups (92). As confirmation of this, aberrancies in the extended motor network and impairments in areas of the temporal cortex associated with language processing are prominent features of ASD (93, 94).

## Neurobiological Aspects Involved in Sex-Specific Behavioral Differences in ASD

### Brain Sex-Related Morphological and Functional Difference in ASD Patients: The Extreme Male Brain Theory

The way by which the sex difference-related male bias is connected to the etiology of ASD has been addressed by researchers (95–97). The extreme male brain (EMB) theory states that specific cognitive and behavioral dimorphisms, linked to the ASD susceptibility, are determined by morphological and functional differences characterizing male and female brains. These include: (i) the total size and volume of the brain (98, 99), (ii) the white/gray matter ratio (100), (iii) the sexual dimorphism of specific brain regions (101, 102), and (iv) the inter/intra-hemispheric connectivity (6, 103, 104). Experimental procedures performed both in humans (105) and in animal models (106, 107) sustain the hypothesis by which, at behavioral level, male brain is focused on “systemizing,” defined as “the ability of analyzing a system and understanding the rules that govern it.” On the contrary, female brain is inclined to excel in “empathizing,” that is the ability to “identify mental states and to respond with appropriate emotions” (108, 109). In sight of this notion, individuals with ASD develop an extreme version of the male brain, thus superior in systemizing performances but unable to empathize (6). A number of studies support this hypothesis: individuals with ASD score higher in systemizing quotient (SQ) parameters (e.g., attention to details, analysis of systems) in respect to neurotypical male individuals who, in turn, score higher than females. In the same way, woman score the best when performing in empathizing quotient (EQ) tests (e.g., Language and empathy) in respect to men, while individuals with ASD score the lowest (6, 110, 111). Morphological evidences seems to support the EMB theory, as boys have larger brains than girls (99) and this difference is more marked in children with ASD (112). The same evidence is recognized in specific brain regions such as the amygdala (113). Despite these evidences, EMB theory loses consistency when specific brain regions from ASD male and female subjects are compared. In particular, a study by the Critchley's laboratory attests that even if sex-related differences can be assessed in total gray and white matter volumes from male and female neurotypical individuals, these differences weaken in individuals with ASD and completely disappear when the investigation of specific brain areas is performed (114). Another study by Lai et al. attests that, at morphological level, a number of brain areas show differences in gray/white matter volumes, when ASD-affected male and female brain is compared with their matched controls. Nevertheless, when the comparison between ASD male and female brains is performed, the overlap between atypical regions is minimal, underlining that distinct dimorphic neuroanatomical features may characterize ASD (87). Although apparently, EMB theory, by which ASD condition is the result of an amplification of the typical sexual dimorphic differences, is fulfilled in ASD females, where structures showing sexual dimorphism display alterations in white/gray matter volumes, this is not true in male ASD. This suggest that even adopting the same behavioral criteria for ASD diagnosis, many neuroanatomical aspects can differ (87). Brain overgrowth and macrocephaly are often observed in ASD diagnosed children (115). Although not directly related to sexual dimorphic differences in normal individuals, affecting particularly frontal and anterior temporal brain regions. This phenomenon is not present at birth (116), but it is observed in 20% of children with ASD at 2 years of age (117, 118). Both girls and boys with ASD show an abnormal enlargement of whole brain and of frontal, temporal and cerebellar white/gray matter volumes, nevertheless girls with ASD display more severely affected temporal white and temporal and cerebellar gray matter, thus showing a significantly greater degree of impairments in respect to boys (79). Despite this result, sample size limitations in female group raise concerns and has to be taken in account. A study by Nordahl et al., involving 180 children of 2–4 age highlighted that macrocephaly was a distinctive sign only in males and no distinguishable alterations were observed in 24 affected females (119).

### Hormonal Differences in ASD Patients

The sexual dimorphism that characterizes the mammalian brain may be explained, at least in part, by developmental differences, by which the gonadal hormones influence is responsible for. In particular, while the female brain develops in relative absence of sexual hormones, starting from the 11 to 13th embryonic day (E) of life in rodents (120) and between 8 and 24th week in humans (121), the primordial testis synthesizes fetal Testosterone (fT), determining the male brain masculinization (122). This hormonal surge produces the structural, behavioral and cognition sex differences characterizing male and female brain (121, 123, 124) acting mainly through the aromatization of Testosterone into 17β-Estradiol (125), although specific androgen receptor-mediated activities are known (126, 127). Consistently, an hypermasculinization effect, induced by increased levels of fT could produce the extreme male brain conditions that lead to the development of autistic traits (6). Different evidences follow these principles: (i) an abnormal increase of fT, determined by an inefficient synthesis of cortisol in the adrenal glands, produces an increase of autistic traits manifestation in females affected, in respect to their typically developing sisters, leading to a genetic condition known as congenital adrenal hyperplasia (CAH) (128). (ii) fT levels are found to be inversely-correlated to the diagnostic behavioral ASD evaluation (i.e., empathy, eye contact, vocabulary development) (129, 130) and directly-correlated to autistic traits, including systemizing quotient and narrow interests (131–134). (iii) The permeability of the placental barrier to the diffusion of testosterone may expose females to ASD susceptibility, although this is not observed in males, where the high embryonic testis-mediated fT synthesis precludes maternal influences. In this scenario, it is important to note that whether prenatal gonadal hormones are the triggering cause of ASD, non-canonical situation in the hormonal profile can persist in post-natal life. Indeed, many medical conditions related to androgens (i.e., acne, hirsutism and Polycystic Ovary Syndrome) are frequently present in women with ASD (135). Furthermore, both Androgen Receptor (AR) and genes controlling Testosterone metabolism are associated with ASD (136–138). Thus, in the search of specific diagnostic biomarkers several studies analyzed hormones' levels in individuals with ASD, in respect to control subjects and between males and females with ASD. However, it should be considered that in addition to Testosterone and Estradiol, two hormonal metabolic intermediates, the dehydroepiandrosterone sulfate (DHEA-S) and the androstenedione, are produced by gonads and adrenal glands, thus contributing to the total systemic levels of active androgens (139). When the complete asset of gonadal and adrenal sex hormones were analyzed in individuals with ASD patients compared to their sex-matched controls, increased levels of androstenedione have been found (140). In line with the EMB theory, an increase of this testosterone precursor could determine high levels of fT during embryonic life, especially in females where the adrenal gland secretion of androstenedione could provide to the lack of the testis-mediated testosterone synthesis. It is worthy to note that no differences in androstenedione levels between male with ASD and female probands were found, although slight fluctuations were observed in females during follicular, ovulatory and luteal phases. Moreover, in support of the EMB hypothesis we report data from a recent meta-analysis on the 2D:4D ratio in various psychiatric district, including ASD. The second-to-fourth digit ratio (2D:4D) is an indirect, retrospective, non-invasive measure that correlates negatively with intrauterine exposure to testosterone. La meta-analysis di Fusar et al. evaluated if 2D:4D differs between patients with psychiatric disorders and controls. They included 43 case-control studies which compared the 2D:4D ratio of patients with ASD spectrum disorder (ASD) (*n* = 16), and other psychiatric disorders. Meta-analyses founded that in the ASD 2D:4D ratio was significantly lower than healthy controls (141). On the contrary, a marked sex-related disparity was observed in testosterone and DHEA-S levels, with increased concentrations in males and higher estradiol expression in females (142). Although the differential secretion of gonadal hormones may explain these findings, the absence of a normal sexual dimorphism as observed in androstenedione synthesis in individuals with ASD, could play a role in ASD susceptibility. In particular, while in males only a small fraction of testosterone derives from the synthesis of the androstenedione, whose major source originates from the testis, in females, 60% of the circulating testosterone comes from the peripheral androstenedione conversion (143). Although is no longer a diagnosis on its own, a marked sexual dimorphism has been reported in individuals with Asperger's syndrome (AS), where differences in the expression of serum hormones and cytokines have been demonstrated (142). In particular, a general upregulation of cytokines and inflammatory molecules (e.g., IL-10, ICAM-1, TNFα, and others), with the only exception of IL-7, was observed in males respect to female, whereas the females with ASD displayed higher amounts of hormones and growth factors (BDNF and Insulin), with the only exception of the Growth Hormone (GH) concentration (142).

#### The Female Protective Effect

Although an environmental influence is hypothesized to take place in ASD etiology (144, 145), a strong genetic component may influence the onset of the disorder, as a 90% heritability has been found in monozygotic twins (146), while only 0–10% was observed in dizygotic twins (147). Single nucleotide mutations and copy number variants (CNV) have been accounted for ASD etiology, although most *de novo* CNV and genetic rare mutations are related with <1% of cases (148). Therefore, rather than a single causative gene, genetic heterogeneity, multiple genes involvement and epigenetic influence (i.e., environmental effects) may concur to the insurgence of ASD. The reduced incidence of ASD in females could be due to specific genetic differences connected to sexual dimorphism. Two models have been proposed to explain this reduced incidence in females: (i) multiple interacting genes, leading to define a threshold of liability higher in females than in males (149). This “Female Protective Effect” (FPE) could explain the more severe prognoses in females with respect to males (13) and the observed reduction of male to female ratio from 4:1 to 2:1 in subjects with severe intellectual disability (8). (ii) an increased penetrance of genes responsible for ASD in males (150, 151). A study from Levy et al. demonstrates that *de novo* CNV affect more genes in ASD females respect to males and suggests that the frequency in autosomal *de novo* CNV is higher in the former (152). The same trend, with more CNV scattered in the females genome, was observed by analyzing the relationship between rare *de novo* CNV and sex (153). Of both these studies suggest that an increased mutational burden is needed in females to develop neurodevelopmental disorders and in particular ASD. Similarly, Eichler et al. demonstrated that, although small and rare CNV (i.e., <400 kb and <1% frequency) equally occur in ASD males and females, CNV larger than 400 kb were 2-fold increased in ASD females, where also the CNV proportion is larger (154), suggesting a greater genetic susceptibility of males than females to develop ASD symptomatology (13).

## Measuring ASD-Relevant Behavioral Male and Female Phenotype in the Laboratory Setting

Success in translational neuroscience will likely require integration of information from diverse model systems along with analysis of human biological samples, large multifaceted human datasets, and human experimental biology. Research performed in laboratory animals is essential to elucidate disease mechanisms because it makes possible functional investigation of disease-associated etiological factors in living brains (155).

Because of their close evolutionary relationship, mice and humans share preservation of genes, biological processes, brain circuitries, and to some extent, behaviors (156). Although ASD is a uniquely human disorder, many of its core deficits can be modeled in rodents through fine behavioral testing (157). Our ability to employ experimental manipulations through genetic engineering and other cutting-edge technologies may not only help us probe the underlying mechanism of the disorder, but may also lead to the development of targeted and effective therapeutic approaches that can later be translated to humans (158). In this context, rodent models have been useful for ASD research in several ways: (1) they present a controllable intact biological system to understand the complex interaction of mutated gene products with other proteins, helping to define convergent molecular pathways that that can later be targeted for treatment; (2) they can be used to define the anatomical and physiological changes in precisely defined microcircuits that may contribute to ASD, helping identify fundamental changes in neural circuitry and biomarkers that may be translated to use in humans, predicting outcome and assessing response to treatment; (3) they would be useful for screening therapeutic effects of behavioral and pharmacological treatments; (4) the advent of models that allow temporally specific genetic deletion and rescue of ASD-related genetic changes would let us to define the critical developmental windows where interventions would be effective; (5) they would be useful in the future for assessing the interaction of specific environmental insults with autism susceptibility genes (158). Besides that, rodent models are able to reproduce sex differences associated to psychiatric disorders in their prevalence, symptomatology and treatment response (76), providing detailed mechanistic information about sex differences in ASD in terms of manifestation, disease progression, and development of therapeutic options (159).

### Male Predominance in Rodent Models of ASD

Animal models of ASD have been mainly developed and validated in male subjects (76). The main reason for this is the diffused assumption that the cyclic variation in female sex hormones may confound the results (160, 161). As a consequence, for years, findings on males were generalized to females, with the ratio of male-only to female-only studies in neuroscience research being around 5:1 (161). Nowadays, there is increasing awareness that sex influences have a profound impact on brain function and new emphasis has been given in recognizing the need of considering gender and sex differences in preclinical studies (162). Thus, despite the majority of preclinical studies in ASD research were performed in males only, few studies are emerging describing the differences in autistic-like traits between male and female animal models.

### Behavioral Sex–Differences in Rodent Models of ASD

In line with the hypothesis that the pathogenesis of ASD is related to environmental and genetic factors, or more likely to a combination of both, the preclinical models of ASD currently available are based on either genetic or environmental factors known to be involved in the pathogenesis of ASD.

### Genetic Rodent Models of ASD

#### Mutation in the Fragile X Mental Retardation 1 (FMR1) Gene

Fragile X Syndrome (FXS) is the most common monogenic form of ASD. The prevalence of the FXS full mutation in the general population is estimated as 1 in 5,000 in males and as 1 in 4,000 to 1 in 8,000 in females (163). The syndrome is associated with an unstable expansion of a CGG trinucleotide repeat within the 5′untranslated region (5′UTR) of the FMR1 gene causing the loss of the Fragile X Mental Retardation Protein (FMRP), a key RNA-binding protein involved in synaptic plasticity and neuronal morphology (164). The prevalence of ASD in FXS patients was reported to be ~50–75% in males and 25% in females (165). Since few years ago, the only animal model of FXS was the Fmr1 knockout (KO) mouse, obtained by the inactivation of the murine gene that causes the loss of FMRP production. Fmr1 KO mice reproduce the major behavioral and synaptic alterations found in FXS patients (166). More recently, thanks to zinc-finger nuclease (ZFN) and CRISPR technologies, Fmr1 KO rats have been generated (167–169). Mutant Fmr1 KO mice and rats display several behavioral alterations which characterize FXS in humans, such as altered social interaction and social play behavior, social anxiety, defects in visual attention and auditory dysfunctions, cognitive deficits, repetitive behaviors and hyperactivity (166, 168–170). To date, only few studies have simultaneously analyzed male and female Fmr1 KO animals. Some studies found no differences between male and homozygous female Fmr1 KO mice at the behavioral level (171, 172). In particular, when tested in tasks exploring spatial learning and memory, both male and homozygous female Fmr1 KO mice exhibited very similar impairments (171). In line with these early findings, both male and homozygous female Fmr1 KO mice displayed impaired contextual and passive avoidance memory, significant audio-genic seizures and hyperactivity in the open field and light–dark tests (172). Conversely, a study performed by Nolan et al. (173) revealed that the deletion of the Fmr1 gene produces sex-specific behavioral changes. In particular, Fmr1 KO homozygous female mice displayed increased repetitive behaviors when tested in the nose-poke test and enhanced motor coordination on the accelerating rotarod compared to wildtype females, whereas a similar effect lacked in Fmr1 KO males which showed hyperactivity in the open field (173). Since the FMR1 gene is located on the X chromosome, when males inherit the X chromosome with the FMR1 mutation from their mother, only the X chromosome is affected (174). Females, instead, which have two X chromosomes, could present a second, “unaffected” X chromosome that allows the production of some FMRP, which is however not sufficient to restore the full FMRP function in most heterozygous females (175). Thus, when autistic-like behaviors were analyzed in heterozygous Fmr1 KO female mice, abnormalities in social interaction and communication were detected at infancy and at the juvenile age (176, 177); at adulthood, some of these alterations disappeared, while avoidance of social novelty appeared, together with hyperactivity and reduced contextual fear response (177).

#### Mutation in the Phosphatase and Tensin Homolog on Chromosome Ten (PTEN) Gene

The PTEN tumor suppressor gene, which encodes a widely expressed phosphatase, was initially identified as a cancer predisposition gene (178, 179). In the last decade, germline mutations in PTEN were discovered as a cause of ASD in children with macrocephaly (180). In particular, the prevalence in PTEN mutation was found to be of 8.3% in pediatric patients with ASD and 12.2% in subjects with developmental delay/mental retardation (181). The PTEN gene seems to have a critical role in the regulation of the phosphoinositide 3-kinase/AKT/mammalian target of rapamycin (PI3K/AKT/mTOR) intracellular pathway, considered to be involved in the behavioral abnormalities that characterize ASD (182). At the preclinical level, conditional PTEN null mice have been generated, leading to a controlled loss of PTEN causing different consequences depending on the cell type or its state of differentiation. Conditional PTEN null mice with loss of the mouse ortholog of the human PTEN gene in neurons of the cortex and hippocampus display autistic-like traits such as reduced reciprocal social interactions, low sociability, impaired nest-building behavior, impaired social recognition (183, 184). Only few studies have been performed in conditional PTEN null female mice. Tilot et al. generated a germline knock-in mouse model of cytoplasm-predominant PTEN (the homozygous PTEN^m3m4^ mice) that displayed sex-specific behavioral deficits in sociability. In particular, PTEN mutant males showed increased social motivation compared to PTEN mutant females and wildtype animals (185). Conversely, other studies found that female PTEN mice are impaired in both the social approach and the social novelty phase of the three chamber task (186, 187) and show altered emotional learning (187). At infancy, sex- and age-specific differences in the acoustic and temporal structures of USVs have been observed in a neuron-subset (NS) specific PTEN KO mouse model (188).

#### Mutations in Neuroligin (NLGN)−3 and−4 Genes

Neuroligins (NLGNs) are essential postsynaptic neuronal cell adhesion molecules contributing to the maturation and function of both glutamatergic and GABAergic synapses. These molecules act with their presynaptic and intracellular binding partners, β-neurexins and SHANK3, respectively (189, 190). Mutations in the Neuroligin (NLGN)-3 and−4 genes have been associated with mental retardation and ASD (191–193) NLGN mutations seem to alter proper synapse maturation during the development of neural circuits shifting the balance between glutamatergic and GABAergic synapses (194, 195). As other models of ASD, differences between male and female NLGN-3 and−4 mutant mice have been poorly investigated. However, evidence exists that sex differences are displayed by this animal model of ASD. Ju et al. reported that NlGN-4 mutant mice show communicative deficits that are more prominent in females (196). A study performed by Kalbassi et al. showed that NLGN-3 KO female mice were insensible to the social environment, and thus to their peers behavior, compared to NLGN-3 KO male mice, which displayed deficits in sociability and social submission together with increased anxiety (197).

#### Mutations in the Tuberous Sclerosis Complex (TSC) 1 and 2 Genes

Mutations in the TSC1 or TSC2 genes cause an extensive neuropathology, leading to ASD features in in 25–50% of patients. TSC patients display the core symptoms of ASD, sometimes associated with seizures, intellectual disability and developmental delay (198). On this basis, mutant mice for the TSC1 or TSC2 genes have been generated. TSC-associated ASD seems to occur in a 1:1 ratio (199, 200) suggesting that similar ASD-like phenotype should be present in males and females. In line with this notion, social impairments seem to be similar in manifestation and magnitude between male and female TSC1 and TSC2 heterozygous mice, reflecting the equal sex ratio in human patients with TSC-associated ASD (201). However, in a novel TSC mouse model based on the specific loss of TSC2 in Purkinje cell, the TSC2f/–Cre mouse, a stronger impairment in social novelty was found in male compared to female mice (202).

#### Heterozygous Mice for the Methylenetetrahydrofolate Reductase (Mthfr)

Defects in Mthfr gene regulation and abnormal homocysteine-folate metabolism have been associated to an increased risk of birth defects such as neural tube defects, oral clefts, and Down syndrome (203). Furthermore, an increased risk has been reported for neuropsychiatric diseases such as depression, obsessive-compulsive disorder, schizophrenia and ASD (204–207). Thus, since Mthfr homozygous mutant mice are not vital, Mthfr heterozygous mutant mice have been proposed as preclinical model of ASD. No sex-differences were found in both male and female Mthfr mutant mice displaying cognitive deficits, hyperactivity, anxiety and low sociability (208, 209). However, when measured in the open field and in social preference tasks, anxiety level and social deficits were higher in females compared to males (208).

#### The Black and Tan Brachyury (BTBR) Mouse Strain

The BTBR T+ tf/J mouse model of ASD is an inbred mouse strain presenting behavioral deficits that mimic the core symptoms of ASD. For instance, BTBR mice show social impairment, repetitive behaviors and an unusual pattern of USVs at infancy and adulthood (210, 211). Few studies have analyzed the behavioral differences between BTBR male and female mice. Coretti et al. reported that female BTBR mice exhibit a specific increase in self-grooming behavior compared to male BTBR mice (212). Furthermore, male but not female BTBR mice expressed higher rates of grooming behavior and locomotor activity compared to control animals (213), revealing gender differences in the expression of restricted, repetitive behaviors. As for the social domain, when tested in three chamber task, male BTBR mice showed social deficits that were not evident in female BTBR mice (214). Conversely, when paired with novel partners of different strains (215), and during female-female interactions (211), BTBR female mice engaged in less social investigation than their male counterpart, indicating that sex-differences in the social behavior displayed by BTBR mice may depend on specific environmental conditions. As for the communicative domain, both male and female BTBR mice showed an unusual pattern of USVs when removed from the mother and siblings (210), revealing an atypical communication that persists in adulthood (211).

### Environmental Models of ASD

According to the multifactorial theory of the etiology of ASD, which postulates a crosstalk between genetic susceptibility and exposure to environmental factors at the basis of the disease, several environmental factors have been correlated with ASD (216). In particular, maternal exposure to several teratogenic agents (such as infections or teratogenic compounds as ethanol, thalidomide, valproic acid, and misoprostol) has been long investigated as a possible cause of ASD (217). The effects of environmental factors on offspring development are strongly related to the gestational time window in which the exposure occurs, with the first trimester of pregnancy being the most susceptible period in humans (218). Based on the clinical findings, different preclinical models of ASD have been conceived that use controlled exposure of laboratory animals to one of the environmental factors involved in the human disease.

#### Prenatal Exposure to Valproic Acid

Valproic acid (VPA) is a widely prescribed medication used for epilepsy and mood disorders. The use of VPA during early pregnancy has been related to several minor and major malformations in the offspring, such as neural tube defects, developmental delay and ASD (219–222). Based on these clinical observations, prenatal VPA exposure in rodents is a widely used environmental preclinical model of ASD with face and construct validity (223, 224). Indeed, studies in both rats and mice confirm that prenatal VPA exposure leads to autistic-like behaviors in the offspring, including social abnormalities, repetitive behaviors and disrupted communication (223, 224). The prevalence of ASD in children exposed to VPA during pregnancy is characterized by a 1:1 male to female ratio (225). In rodents, prenatal exposure to VPA induces autistic-like behaviors in both the male and female offspring (166, 226, 227), although these deficits appear more pronounced in males compared to females (166, 228). As an example, increased electric-shock induced seizure susceptibility, reduced pain sensitivity and increased anxiety-like behaviors were observed in VPA-exposed male rats but not in their female littermates (228–230). However, increased repetitive/stereotyped behaviors were observed in both male and female VPA-exposed rats together with similar abnormalities in the visuospatial attention and sensorimotor gating behaviors (166, 228, 231). Concerning the social domain, controversial results have been reported. Some authors reported aberrant social behaviors only in male rats prenatally exposed to VPA (228, 229), while some others recognized milder (166, 230) or similar (231, 232) social impairments in VPA-exposed female compared to male animals. The differences described could be due to the rat strain, the behavioral paradigms used and to the VPA dose administered during pregnancy. It is possible that VPA exposure may induce age-dependent social deficits in the female offspring, as for example VPA-exposed females showed atypical patterns of social play behavior at adolescence like their male counterpart, although they showed normal sociability in the three-chamber test at adulthood (166). Similar results were obtained in mice, as for example both male and female VPA-exposed animals exhibited anxiety-like behaviors and memory deficits, with social interaction deficits limited only to male mice (227).

#### Rodent Models Based on Maternal Infection

Epidemiological studies in humans have provided substantial evidence that prenatal infection is associated with an increased risk for the development of several psychiatric disorders, including ASD. The consequences of maternal infection on the offspring are highly dependent on the stage of fetal development at the time of the infection: the fetus seems to be more susceptible to viral infections in the first trimester of pregnancy, while bacterial infections seem to be more problematic in the second trimester (233). Rather than the involvement of direct central nervous system infection, it is more likely that an alteration of the immune system of the mother or offspring act as a trigger event capable to induce ASD (234). Indeed, the activation of the maternal immune system after exposure to viruses and bacteria during pregnancy induces cytokine release able to cross the placenta and alter fetal brain development (235). In line with epidemiological data, preclinical studies performed in rodents have shown that maternal infection induces autistic-like symptoms in the offspring. To date, the most used rodent models of ASD based on maternal immune activation (MIA) employ immunogens such as lipopolysaccharide (LPS) and polycytidylic acid (poly I:C) to mimic bacterial and viral infections, respectively (236). Injection of poly I:C on GD 9.5 or 12.5 causes impairments in social interaction, anxiety and repetitive behaviors in the offspring (237). Similarly, LPS exposure on GD 9.5 resulted in social deficits, communication abnormalities and cognitive inflexibility (238). Few studies analyzed sex-differences in the MIA models of ASD. For example, prenatal exposure to LPS was found to produce pronounced hyper-sensitivity to acoustic startle stimuli in male, but not in female rats (239). In mice, both male and female MIA-exposed animals were found to display sex-specific behavioral ASD-like impairments (240–242). Interestingly, in the three-chamber test, female mice prenatally exposed to both poly (I:C) and LPS showed a reduction in social preference and displayed no stereotypies, while males had social dysfunctions when exposed to prenatal poly (I:C) but not to LPS, and they displayed stereotypies (240). On the contrary, in another study, maternal poly (I:C) exposure reduced social interaction and increase grooming behavior in male but not in female exposed mice (241). Nevertheless, another study revealed that prenatal exposure to poly (I:C) impaired social interaction and increased marble burying in both the male and female offspring, whereas increased anxiety and decreased pre-pulse inhibition were observed only in males (242). Furthermore, Schwartzer et al. failed to detect pronounced sex-specific effects of MIA exposure in ASD-relevant behaviors (243). Again, these controversial findings could be explained by the variability in dosage and timing of immunogen injection during pregnancy.

## Concluding Remarks

Although the most recent epidemiological studies have revised downwards the higher prevalence of ASD in males compared to females from 4:1 to 3:1, the data of the highest prevalence in male compared to females is well-documented (4). In addition, several studies are increasingly confirming the specificity of sex differences in clinical phenotype (47). This justifies the need to better understand the causes of this difference and to experimentally test theoretical models such as Extreme Male Brain (244), Female Protective Effect (7–9), and Female Autism Phenotype (10–12). The differences in phenotypic manifestations suggest the need to deepen the sex variability at neurobiological, brain imaging and laboratory level. This may allow an adaptation of tools and methods for identification, evaluation and intervention based on sex differences. In the specific, the imaging remains a powerful tool to see subtle differences in brain structure. In particular, MRI techniques are among the most promising non-invasive tools for investigating the neurological underpinnings of ASD, which are essential for developing discriminative neuroimaging biomarkers for clinical diagnosis with the potential to inform precision psychiatry. Despite the difficulty of having a large number of girls in the ASD group, the study of Supekar et al. suggested to integrate three publicly available data-sets to address this problem (92). While, it is also important that preclinical studies with animal models take serious account of the sex/ difference in ASD. Being a neurodevelopmental disorder, ASD is notoriously difficult to model in laboratory animals. Indeed, since the exact etiology of ASD is unknown and given the variability in the phenotypic presentation of its core and comorbid symptoms, generating an animal model able to capture at once all the facets of ASD is far from simple. In the last decade, a number of rodent models of ASD have been generated, able to reproduce at least the core features of the pathology with a certain reproducibility in male animals (157, 177, 245). On the other hand, although it is now accepted that preclinical studies should include female as well as male subjects, the behavioral characterization of female rodent models of ASD is still at the beginning. Some studies that included female subjects reported controversial results and sex differences were not always evident [i.e., (171, 172, 201, 209)], thus failing in reporting the established sex dimorphism displayed by ASD patients (65, 246). This may be due to different reasons, such as the rodent model used (i.e., the specific mutation or the environmental factor used to mimic the disease), the different ASD male to female ratio caused by the chosen mutation or environmental factor (i.e., VPA-associated ASD seems to occur in a 1:1 ratio (225) compared to the 3:1 ratio of ASD in the general population (4) or the behavioral tasks used to assess ASD-like features. Thus, there is the possibility that, in addition to the canonical behavioral paradigms used to detect the core symptoms of ASD, other tasks should be used to assess the female autistic phenotype in laboratory animals, in order to detect subtler symptoms often reported by female ASD patients such as depression, anxiety and emotional changes (15, 73, 74, 247). However, other studies including female rodents revealed a substantial amount of sex-related differences in several behavioral tasks commonly used to assess core and comorbid autistic-like features (166, 180, 212, 213, 228). Taken together, these considerations underscore the need to include female subjects in clinical and preclinical studies with specifically targeted assessment tools. Moreover, studying sex-dependent behaviors such as sociability or emotional reactivity could be a novel approach to reveal ASD sex-dimorphic behavioral features that will help to shed light on the underlying mechanisms at the bases of the disorder and even to further improve the identification, evaluation and intervention in ASD based on sex differences.

## Author Contributions

AN, SS, PL, MR, and SV contributed to conception and design of the study. SP, FB, EL, and VT collected related literature and tabulated it. FB, EL, ET, LC, and FP wrote sections of the manuscript. All authors contributed to manuscript revision, read, and approved the submitted version.

## Conflict of Interest

The authors declare that the research was conducted in the absence of any commercial or financial relationships that could be construed as a potential conflict of interest.

## Publisher's Note

All claims expressed in this article are solely those of the authors and do not necessarily represent those of their affiliated organizations, or those of the publisher, the editors and the reviewers. Any product that may be evaluated in this article, or claim that may be made by its manufacturer, is not guaranteed or endorsed by the publisher.
